# The Practice of Medicine

**Published:** 1904-08

**Authors:** 


### The Practice of Medicine.

The practice of medicine is variously defined, but the most limited application of the term is made by those who wish to practice medicine without complying with the reasonable requirements of law. The profession is always glad to see judges look at the matter in a broad way, as Judge Davis of the Court of Quarter Sessions of Philadelphia has done. His charge to the jury in the case of one Thomas E. Eldridge contains a clear and concise statement that is worth quoting:

*First.*—The practice of medicine consists in the offering of service and assuming the responsibilities of treating diseases, deformities and injuries, no matter by what means this is professed to be done.

*Second.*—Every State, because of the inherent nature of the calling of medicine, possesses the right both constitutionally and legally to demand a standard degree of qualification which will protect citizens from the consequences of incompetency and unskilled practice.

*Third.*—Anyone practicing medicine without the license of the State, which license is on the part of the State a guarantee of the possession of the qualification to safely pursue medical practice, is an illegal practitioner.

Everyone, therefore, who offers service as outlined, no matter by what means he professes to treat diseases, deformities and injuries, must, as a condition precedent thereto, have obtained the license in accordance with the laws of the commonwealth as finds them so doing.

This makes the practice of medicine—so far as the jurisdiction of the court in question is concerned—consist of the offering of service as a physician and the assumption of the responsibility of treating disease. The means used is not a matter of vital importance and the attempted evasion of the law by the defense that the alleged doctor did not prescribe drugs, etc., was seen to be a fallacy by the impartial judge. The trial developed the fact that the defendant had a diploma from an institution called the Eastern College of Electro-Therapeutics, and that he was practicing on the basis of that authority, pretending to be exempt from the medical practice law of the State of Pennsylvania. This calls attention again to some institutions which, although they pretend to give regular courses of instruction in certain branches, are little better than diploma mills, in that they turn out half-educated men who are prejudiced against the science of medicine in its broad sense, and who are ready to evade the laws of the State wherever they require more education than these would-be doctors possess.—*Journal A. M. A.*

[The courts of several States have also decided as above, notably Missouri, Alabama, and Illinois.—Ed.]

